# Premature Myocardial Infarction: Genetic Variations in SIRT1 Affect Disease Susceptibility

**DOI:** 10.1155/2019/8921806

**Published:** 2019-04-15

**Authors:** Aylin Hatice Yamac, Omer Uysal, Ziya Ismailoglu, Mehmet Ertürk, Mert Celikten, Ahmet Bacaksiz, Ulkan Kilic

**Affiliations:** ^1^Bezmialem Vakif University, Faculty of Medicine, Department of Cardiology, Istanbul, Turkey; ^2^Bezmialem Vakif University, Faculty of Medicine, Department of Biostatistics, Istanbul, Turkey; ^3^University of Health Science, Mehmet Akif Ersoy Heart Hospital, Department of Cardiology, Istanbul, Turkey; ^4^Bezmialem Vakif University, Research Center, Istanbul, Turkey; ^5^University of Health Science, Faculty of Medicine, Department of Medical Biology, Istanbul, Turkey

## Abstract

**Objectives:**

Premature myocardial infarction (PMI) is an uncommon disease, and its incidence varies between 2% and 10%, rising, depending on genetic susceptibility under the influence of lifestyle. The purpose of this study was to investigate the association between *SIRT1* single nucleotide polymorphisms (SNPs), SIRT1, and eNOS (endothelial nitric oxide synthase) protein expressions, total antioxidant status (TAS), total oxidant status (TOS), and oxidative stress index (OSI) in young patients with premature ST-elevation myocardial infarction (STEMI).

**Methods:**

Genotyping of the three single-nucleotide polymorphisms (rs7895833 A > G in the promoter region, rs7069102 C > G in intron 4, and rs2273773 C > T in exon 5) in *SIRT1* gene was performed in 108 consecutive patients (87.0% were men with a mean age of 40.74 ± 3.82 years) suffering from ST-elevation myocardial infarction at the age of ≤45 and 91 control subjects.

**Results:**

The risk for myocardial infarction was increased by 2.31 times in carriers of CC or CG genotypes. SIRT1 protein levels were enhanced and endothelial nitric oxide synthase levels were diminished in ST-elevation myocardial infarction patients regardless of the underlying gene variant. There was no correlation between SIRT1 expression and the amount of endothelial nitric oxide synthase, total antioxidant status, total oxidant status, and oxidative stress index levels in patients and in the control group either.

**Conclusions:**

*SIRT1* single-nucleotide polymorphisms were associated with premature myocardial infarction, which affected the SIRT1 and endothelial nitric oxide synthase protein expression, irrespective of the underlying *SIRT1* genotype.

## 1. Introduction

Myocardial infarction (MI) is an atypical disease in young individuals with a varying prevalence between 2% and 10% [1–6].

An increasing prevalence of risk factors for coronary artery disease (CAD), such as impaired glucose tolerance and obesity in adolescence, is counteracting for the protection featured by young age [7]. Thus, in this population, an early recognition and a risk factor modification is of key importance [8].

Pathogenesis of myocardial infarction in young people is likely to be different from those in an older population [9]. Patients with premature myocardial infarction (PMI) tending to present fewer diffuse atherosclerotic coronary arteries are mostly indicated by a single vessel disease [10–12] and generally have a good outcome comparing to older patients [4, 13]. Most of the cardiovascular risk factors and accordingly the development and survey of cardiac disease have a predominant genetic component. Identifying the effect of these possible genetic modifiers on disease phenotype might be of great interest to detect young people at risk very early.

It has been shown before that some of the *SIRT1* single-nucleotide polymorphisms (SNPs) are associated with obesity, disturbed glucose tolerance and diabetes, arterial hypertension, dyslipidemia, and coronary artery sclerosis, which may shape the cardiovascular phenotype [14–18]. In the present study, three candidate single-nucleotide polymorphisms of the *SIRT1* gene (rs7895833 A > G in the promoter region, rs7069102 C > G in intron 4, and rs2273773 C > T in exon 5 silent mutation) were investigated related to the pathogenesis of coronary artery disease through modifying SIRT1 protein expression [19].

The *SIRT1* gene is located on chromosome 10q21.3 (PUBMED gene and composed of 11 exons and 10 introns, updated on 30 November 2014) [20]. Seven different sirtuin (SIRT) proteins in humans (SIRT1-7) are known to possess cardioprotective properties, including the regulation of vascular endothelial homeostasis, angiogenesis, endothelial senescence, and endothelial function through the modulation of endothelial nitric oxide synthase activity [20–23]. The resistance of myoblasts to oxidative stress is mediated by SIRT1 by enhancing the activity mangan superoxide dismutase (MnSOD) [24], whereas overexpression of SIRT1 protein diminishes oxidative stress by activation of FoxO1-dependent pathway [25], latter resulting in reduced cardiac infarct volume and improved functional recovery after ischemia/reperfusion in mice [26].

The purpose of this study was to investigate the association between *SIRT1* single-nucleotide polymorphisms (rs7895833 A > G in the promoter region, rs7069102 C > G in intron 4, and rs2273773 C > T in exon 5 silent mutation) and the levels of SIRT1 and endothelial nitric oxide synthase expression, total antioxidant status (TAS), total oxidant status (TOS), oxidative stress index (OSI) as well as common cardiovascular risk factors and major adverse cardiac events (MACEs) in young patients suffering from premature ST-elevation myocardial infarction (STEMI).

## 2. Methods

### 2.1. Study Groups

Patients included in this study presented with a history of premature myocardial infarction (PMI) to the Departments of Cardiology of the Bezmialem Vakif University Hospital and Mehmet Akif Ersoy Heart Hospital between January 2012 and May 2015. Patients with malignancies, major trauma or surgery in the previous six months, and acute or chronic infectious disease were excluded. The randomly selected healthy control subjects were selected from people, who came to Bezmialem Vakif University Hospital for a routine examination without known cardiovascular diseases. According to the power analysis (80% power with a 95% confidence interval), the study groups consisted of 108 patients, who had suffered from a premature ST-elevation myocardial infarction under the age of 45 (87.0% men, mean age 40.74 ± 3.82 years) and 91 control subjects (57.1% men, mean age 32.7 ± 6.3 years, *p* < 0.001). The difference of SIRT1 protein between both groups (STEMI vs control) was at least 0.7 ng/ml for any subgenotype with a standard deviation of 1.7. Corresponding to the power analysis, a minimum of 94 patients in the STEMI group were necessary to calculate a significant difference.

The control subjects were healthy patients without a known cardiovascular disease or other significant comorbidities like liver, kidney, lung, or oncologic disease.

The Ethical Committee of the Faculty of Medicine at the Bezmialem Vakif University Hospital approved the study. All participants gave a written informed consent and completed a structured questionnaire including their demographic data. The ethical principles described by the Declaration of Helsinki were exerted.

### 2.2. Biochemical and Demographic Analysis

After 12 hours of fasting, whole blood samples were taken and transferred into plain tubes (Vacuette, Greiner Labor technic, Germany). After centrifugation for 5 min at 4,500 rpm at +4°C and removal of serum and plasma, samples were stored at −20°C. Parameters like fasting glucose, total cholesterol, triglyceride, high-density lipoprotein (HDL) cholesterol, low-density lipoprotein (LDL) cholesterol, and very low-density lipoprotein (VLDL) were determined in all patient samples. Body mass index (BMI) was calculated by dividing weight by height square (kg/m^2^).

### 2.3. Determination of SIRT1 and Endothelial Nitric Oxide Synthase Protein Levels

Enzyme-linked immunosorbent assay (ELISA) kits from USCN Life Science (Catalog no.: E94912Hu for SIRT1 and SEA868Hu for eNOS, Wuhan/CHINA) were used to determine levels of SIRT1 and endothelial nitric oxide synthase proteins.

After incubation of the samples with antibody-coated 96-well plates, enzyme-linked antibodies for the proteins were added. The intensity of the color was measured in a microplate reader (Chromate Manager 4300, Palm City, USA) at 450 nm.

### 2.4. Measurement of Total Antioxidant and Oxidant Status

The measurements of the total antioxidant status (TAS) and the total oxidant status (TOS) were conducted by a microplate reader (Chromate Manager 4300, Palm City, USA) as described previously [19] using an automated measurement method (Rel Assay Diagnostics, Gaziantep/TURKEY) [27, 28]. Measured values were given in mmol equiv/L trolox. The oxidative stress index (OSI) was determined as OSI = ((TOS)/(TAS × 1000)) × 100 [19].

### 2.5. DNA Isolation

The genomic DNA was isolated from peripheral blood leukocytes with the DNA isolation kit (Invitrogen, Carlsbad, USA). Thereafter, DNA samples were stored at +4°C and PCR analysis was performed [29].

### 2.6. Determination of SIRT1 Gene Variants

The PCR-CTPP technique was used to analyze the gene polymorphisms of *SIRT1* rs7895833 A > G in the promoter region, rs7069102 C > G in intron 4, and rs2273773 C > T in exon 5 [19, 30]. The technique is described in detail in our previous publication [19].

Three genotypes for each polymorphism were defined as the following patterns [19]: for rs7895833 A > G polymorphism: 320 and 241 bp for AA genotype; 320, 241, and 136 bp for AG genotype; and 320 and 136 bp for GG genotype; for rs7069102 C > G polymorphism: 391 and 277 bp for CC genotype; 391, 277, and 167 bp for CG genotype; and 391 and 167 bp for GG genotype; for rs2273773 C > T polymorphism: 314 and 228 bp for CC genotype; 314, 228, and 135 bp for CT genotype; and 314 and 135 bp for TT genotype [19].

### 2.7. Definition of Major Adverse Cardiac Events (MACEs) and Analysis of Synthax Score

Major adverse cardiac events (MACEs) included recurrent nonfatal myocardial infarction, hospitalization due to decompensated heart failure, and target vessel revascularization (TVR). In addition, the synergy between PCI with Taxus and cardiac surgery (SYNTAX) score was applied to all coronary lesions with a diameter stenosis greater than 50% in vessels with a diameter larger than 1.5 mm as described before [31].

### 2.8. Echocardiography

The electrocardiogram-guided echocardiographic examination, using a transthoracic approach, was performed by an experienced sonographer who was blinded to the study in terms of infarction type, date, and genetic variation, with the patient in the left lateral decubitus position within 48 hours after admission and 1 year after MI, by using the Philips envisor C HD (Philips Medical Systems, Andover, MA, USA) echocardiography devices and 2–4 MHz phase transducers. All measurements were obtained as per the criteria recommended by the current guidelines [32]. Left ventricular ejection fraction (LVEF) from apical four- and two-chamber views was measured by using the modified Simpson method [33].

### 2.9. Statistical Evaluation

All continuous variables were tested for normality using the Kolmogorov–Smirnov test. Data are presented as percentages, mean ± standard deviation (SD), or median (interquartile range). Chi-squared analysis was used for comparing categorical variables between groups like the distribution differences of the genotypes or alleles in *SIRT1* gene between experimental and control groups.

Differences in continuous variables were tested with the Student's *t*-test or Whitney *U*-test for parametric and nonmetric variables, respectively. Statistical analysis was performed using SPSS version 2.0 (SPPS Inc., Chicago, IL, USA). Relative risk at 95% confidence intervals (CI) was given as the odds ratio (OR). Pearson's correlation test was also used to determine the relation between parameters of interest. All tests were two sided, and *p* < 0.05 was regarded as significant.

## 3. Results

### 3.1. Patients' Demographic and Biochemical Analysis

The clinical characteristics of the study groups are given in Table 1. Fifty-five patients had a ST-elevation myocardial infarction under the age of 40.

Patients suffering from premature ST-elevation myocardial infarction were more likely to be overweight (BMI >25 : 79.1% vs 45.1%, *p*=0.001), with a higher rate of smokers (87.1% vs 58.0%, *p*=0.001), and had dyslipidemia (73.1% vs 12.1%, *p* < 0.001) compared to the control group. The prevalence of diabetes mellitus (18.5% vs 3.3%, *p*=0.001) and hypertension (37.0% vs 2.2%, *p* < 0.001) were significantly higher in the ST-elevation myocardial infarction group. The concentrations of serum triglycerides (*p*=0.003), very low-density lipoprotein (VLDL) (*p*=0.026), fasting blood glucose (*p* < 0.001), and uric acid (*p* < 0.001) were significantly enhanced in ST-elevation myocardial infarction patients, while high-density lipoprotein (HDL) levels (*p* < 0.001) were decreased (Table 2). The LDL levels were comparably high in both groups, taking into account that most of the ST-elevation myocardial infarction patients were under statin therapy.

Anterior myocardial infarction (MI) was more apparent than other myocardial infarction types (51.9%). Seventeen patients underwent percutaneous coronary intervention (PCI) at follow-up (FU), among them 12 with recurrent myocardial infarction, whereas 3 patients were hospitalized due to decompensated heart failure. After myocardial infarction, left ventricular ejection fraction (LVEF) was slightly decreased with an average of 51.9 ± 9.2%, while twenty-five patients had a left ventricular ejection fraction less than 45% (Table 1).

### 3.2. Relationship between Expression Levels of SIRT1 and Endothelial Nitric Oxide Synthase and Levels of Total Antioxidant Status, Total Oxidant Status, and Oxidative Stress Index

SIRT1 protein levels were significantly increased in patients as compared to control subjects (*p* < 0.001). To investigate the role of SIRT1 protein in premature myocardial infarction, other associated parameters such as levels of endothelial nitric oxide synthase and oxidative stress markers and their relation with SIRT1 protein were analyzed.

In contrast to SIRT1 protein, levels of endothelial nitric oxide synthase protein were lower in the patient's group (*p*=0.001) (Table 3).

A positive correlation was detected between SIRT1 expression and age (*p*=0.026) in patients but not in controls (Table 4). In contrast, there was no correlation between SIRT1 expression with the levels of endothelial nitric oxide synthase, total antioxidant status, total oxidant status, and oxidative stress index in patients and in the control group either (*p* > 0.05) (Table 4).

### 3.3. Frequencies of *SIRT1* (rs7895833 A > G, rs7069102 C > G, and rs2273773 C > T) Gene Polymorphisms

The frequencies of genotypes and alleles of *SIRT1* gene in both groups are given in Table 5.

For rs7895833 A > G in the promoter region, only the wild-type AA genotype and heterozygote mutant AG genotype were detected, indicating no difference in distribution of genotypes and alleles between patients and control group (*p* > 0.05).

For rs2273773 C > T in exon 5, all the members of the study population were carrying the heterozygote mutant CT genotype. For rs7069102 C > G in intron 4, the frequency of CG genotype was significantly higher in ST-elevation myocardial infarction patients compared to that of the control group (*p*=0.027). The C allele was predominantly found in the patients group, nearly significant (*p*=0.06) (Table 5).

The risk for myocardial infarction was increased by 2.31 times in carriers of CC or CG genotypes (*χ*^2^: 7.046, *p*=0.008, OR: 2.31, 95% Cl: 1.24–4.31).

### 3.4. Relationship between Protein Levels of SIRT1 and Endothelial Nitric Oxide Synthase and *SIRT1* Gene Polymorphisms

For the defined SNPs, distributions of genotypes and their associations with SIRT1 and endothelial nitric oxide synthase levels are shown in Table 6.

In patients with the SNP rs7895833 A > G, SIRT1 protein levels were upregulated and endothelial nitric oxide synthase expression was markedly decreased in the collective carrying a wild-type (AA) and heterozygote mutant (AG) genotype as compared to that od=f the control group (*p*=0.001 vs *p*=0.031; *p*=0.002 vs *p*=0.002), while there was no difference in the frequency of gene variations between the patient and control groups (*p* > 0.05) (Table 5).

In patients with the SNP rs7069102 C > G SIRT 1, protein expression was markedly upregulated in the collective carrying the homozygote wild-type genotype (CC) (*p*=0.004), while there was no statistically difference in endothelial nitric oxide synthase expression between the patient and control groups (*p* > 0.05). For the CG and GG genotypes, SIRT1 levels in the patients' group were significantly higher, while endothelial nitric oxide synthase levels were markedly decreased compared to those of the control group (*p*=0.002 vs *p*=0.024; *p*=0.006 vs *p*=0.014) (Table 6). Parallel to this, the distribution frequency of the heterozygote CG genotype and moreover of the C allele was higher in the patient group (Table 5).

### 3.5. Relationship between Cardiovascular Risk Factors, Disease Survey, *SIRT1* Gene Variants, and SIRT1 and Endothelial Nitric Oxide Synthase Proteins

If the cardiac risk factors were analyzed separately in each genotype, there were some significant differences between the groups (Table 7). High-density lipoprotein (HDL) levels were decreased in ST-elevation myocardial infarction patients regardless of the genotype. The amount of low-density lipoprotein (LDL) was comparable in the ST-elevation myocardial infarction and control group without a significant difference. Thereby, it should be considered that most of the patients with a history of ST-elevation myocardial infarction were taking statins.

Among patients carrying the rs7069102 C > G CC genotype, only high-density lipoprotein levels were decreased without significant differences in the distribution of the other risk factors compared to the control group (*p*=0.012). In contrast, patients with the heterozygote CG and the homozygote mutant GG genotypes were characterized by enhanced body mass index (*p* < 0.001; *p* < 0.001), elevated levels of triglycerids (*p*=0.005; *p*=0.016), very low-density lipoprotein (VLDL) (*p*=0.008; *p*=0.021), and triglycerids/high-density lipoprotein (*p*=0.001; *p*=0.003). Moreover, levels of uric acid and fasting blood glucose were increased in patients carrying the heterozygote genotype compared to the control collective (*p*=0.002; *p*=0.007).

Among patients with the rs7895833 AA genotype, triglycerids, very low-density lipoprotein, uric acid levels, and body mass index were enhanced (*p* = 0.021, 0.044, 0.003, and <0.001), while the high-density lipoprotein levels were decreased (*p* < 0.001). In the patients with AG genotype, very low-density lipoprotein levels were statistically not relevant (*p*=0.183), but the fasting blood glucose was higher compared to that of the control group (*p*=0.001). The other parameters were equally distributed as in the AA group (Table 7).

Baseline fasting blood glucose concentration was significantly higher in the ST-elevation myocardial infarction group compared to that of the control group, suggesting that a higher proportion of patients had undetected diabetics or prediabetes. SIRT1 protein levels were similarly increased (*p*=0.009; *p* < 0.001), and endothelial nitric oxide synthase levels were similarly decreased (*p*=0.018; *p*=0.005) in diabetic and nondiabetic patients compared to the control group. There was no difference in rs7069102 C > G genotype distribution between diabetic and nondiabetic patients (*p*=0.084).

Patients with the heterozygote rs7069102 CG genotype tended to undergo anterior myocardial infarction (71.4%) more frequently than other myocardial infarction types (61.5%) (*p*=0.068, nearly significant) without differences in SIRT1 and endothelial nitric oxide synthase expression between both groups. Patients carrying the rs7895833 AA genotype possessed higher SYNTAX score than patients carrying the AG genotype (*p*=0.04). There was no difference in SYNTAX score values between the three genotypes of the rs7069102 single-nucleotide polymorphism (*p*=0.548). All patients with recurrent myocardial infarction had their first coronary event under the age of 40. These patients had higher levels of SIRT1 protein (*p*=0.026) compared to patients without an adverse event, while endothelial nitric oxide synthase levels were similar. There was no difference in SNP distribution. We detected a negative correlation between recurrent myocardial infarction and left ventricular ejection fraction, (*r*=−0.233; *p*=0.033) as well as between SIRT1 values and left ventricular ejection fraction (*r*=−0.162; *p*=0.029).

## 4. Discussion

The purpose of this study was to investigate the association between *SIRT1* single nucleotide polymorphisms (rs7895833 A > G in the promoter region, rs7069102 C > G in intron 4, and rs2273773 C > T in exon 5) and development of premature ST-elevation myocardial infarction ≤45 years. Levels of SIRT1 and endothelial nitric oxide synthase proteins, total antioxidant status, total oxidant status, and oxidative stress index were measured to investigate the relationship between genetic variation and phenotype; further, we investigated the association between protein expression and cardiovascular risk factor constellation.

SIRT1 protein levels were markedly increased in myocardial infarction patients compared to that of the control group (Table 3). This result confirmed the findings of our previous study, showing that patients with chronic coronary artery disease were marked by higher levels of SIRT1 compared to the control group [19, 34].

Hereby, the increase in SIRT1 levels was explained as a compensatory mechanism to induce the production of antioxidants against oxidative stress in patients with cardiovascular disease. Kaneda et al. [35] had showed that plasma levels of advanced oxidation protein products (AOPP) were significantly higher in patients with coronary artery disease than in those without. According to the Gensini scoring system, levels of advanced oxidation protein products correlated with severity score of coronary artery disease. However, in the present study, patients with ST-elevation myocardial infarction were mostly characterized by a single vessel disease without diffuse atherosclerosis and without ongoing ischemia after successful percutaneous coronary intervention. Most of the patients recovered with likewise acceptable left ventricular ejection fraction. Thus, they were not confronted with ongoing oxidative stress, yielding normal total antioxidant status, total oxidant status, and oxidative stress index levels.

Endothelial nitric oxide synthase levels were determined in order to investigate the relationship between SIRT1 and endothelial function. In young adults, cardiovascular disease risk factors promote endothelial dysfunction, while in older individuals advancing age per se is independently associated with the development of vascular endothelial dysfunction. This endothelial dysfunction is a result of decreased nitric oxide synthesis, paralleled by increased endothelial oxidative stress and inflammation, which can be also augmented by cardiovascular disease risk factors in older adults. Moreover, lack of endothelial nitric oxide synthase is associated with different disease constellations like hypertension, ventricular hypertrophy, and diet-induced atherosclerosis [36–39].

In our study, the level of endothelial nitric oxide synthase protein was considerably low in ST-elevation myocardial infarction patients compared to those of the control group (Table 3), whereas no correlation was found between levels of SIRT1 and endothelial nitric oxide synthase (Table 4).

Thus, the decreased levels of eNOS and increased levels of SIRT 1 might be disease specific but regulated via different mechanisms.

When we analyze the MI susceptibility of the genotype variants, our results revealed that carrying the CC or CG genotypes, additionally the wild-type C allele of the rs7069102 single-nucleotide polymorphism, was associated with an increased myocardial infarction risk. SIRT1 levels were markedly elevated in the ST-elevation myocardial infarction group, irrespective of the underlying genotype (Table 6). On the other hand, endothelial nitric oxide synthase levels were decreased in all genotypes of rs7895833 and rs7069102, except in rs7069102 CC, where no statistically relevant difference was observed compared to the control group, possibly due to the low subject numbers in this group (Table 6). SIRT1 protein levels for all studied single nucleotide polymorphisms were significantly increased in patients carrying heterozygote mutant genotypes and also mutant genotype for rs7069102 C > G as compared to the control group.

However, in the previous study, the risk for cardiovascular disease was increased by 2.4 times for rs7069102 C > G and 1.9 times for rs2273773 C > T in carriers of mutant allele compared to carriers of wild-type allele (*p* < 0.001), pointing out the protective role of C allele for both single-nucleotide polymorphisms against cardiovascular disease [19]. How can we explain this difference between both studies?

In the first study, patients were older, had undergoing symptoms, and were characterized by diffuse coronary artery disease pathology.

In the current study, patients had suffered from an acute disease, were symptom-free, and were mostly characterized by a single vessel disease with thrombotic occlusion, rather than diffuse atherosclerosis. Thus, the function of *SIRT1* single-nucleotide polymorphisms may vary in these two different disease types. The difference in disease pathogenesis is suggested to be the reason, why in patients with stable coronary artery disease the rs7069102 G allele was associated with risk [19], whereas in premature myocardial infarction the C allele was determined as the risky allele. Besides, another study revealed an association between haplotype rs7069102G-rs3818292A-rs4746720T containing the rs7069102 G allele of *SIRT1* gene and the risk of myocardial infarction development in a Chinese population, including patients of a wide age range with most of the patients being older tan 40 years [40].

An important observation for age- and/or disease-dependent changes in gene polymorphism is as follows: the GG genotype of the rs7895833 A > G was neither present in obese children nor in young adults suffering from ST-elevation myocardial infarction, whereas at the later age, it appeared frequently in people suffering from chronic coronary artery disease [19, 41]. On the other hand, in healthy children, adults, and old people without a cardiovascular disease, the GG genotype was not found [34]. For rs2273773 C > T, only the CT genotype was found in both obese and normal weight children and in our young study collective, while the other genotypes became present at the later age [19, 34, 41].

Finally, we investigated the relationship between gene variants, protein expression, and disease characteristics:

Interestingly, our patients had a high cardiovascular risk profile, including diabetes (18.5% vs 3.3% *p*=0.001), smoking history (87.1% vs 63.8% *p*=0.727), and dislipidemia (73.1% vs 12.1% *p* < 0.001). Uric acid (UA) levels were elevated in the myocardial infarction group (Table 2), and they were correlating positively with endothelial nitric oxide synthase levels (*r*=0.401, *p* < 0.001). Many experimental and epidemiological data have suggested a possible role for hyperuricemia in inducing endothelial dysfunction and particularly impaired nitric oxide (NO) bioavailability, showing a causal role for UA in the development of coronary heart disease or death from cardiovascular causes [42]. Lack of nitric oxide may result in an increased induction of endothelial nitric oxide synthase to compensate the nitric oxide deficiency.

Patients with the genotype rs7069102 CC seemed to have few risk factors compared to all other genotypes, only marked by reduced high-density lipoprotein levels compared to those of the control group. On the other sight, if the G allele came into the game, the risk factor distribution was similar to the whole myocardial infarction collective with an increased risk factor constellation (Table 7).

This finding seemed to be controversial at the first view, as the C allele was defined as the allele increasing the susceptibility for premature myocardial infarction. Thereby, it should be considered that most of our patients (*n*=72) were carrying the CG genotype, while only a few patients (*n*=12) were carrying CC genotype. Thus, we might assume that the risk factor constellation would be equalized between the groups, if more patients would be included in the CC group.

Besides the fact that anterior myocardial infarction was more common compared with other myocardial infarction types. Most of these patients were carrying the rs7069102 CG genotype (*p*=0.068). There was no difference in SIRT1 and endothelial nitric oxide synthase expression between patients with anterior myocardial infarction and other myocardial infarction types (*p*=0.602; *p*=0.984). There was no statistically difference in SYNTAX score values between the genotype variants of the rs102 SNP (*p*=0.548), while SYNTAX score was higher in patients carrying the rs AA genotype compared to that of the AG genotype (*p*=0.04).

All patients with recurrent myocardial infarction had their first coronary event under the age of 40. They had higher levels of SIRT1 protein (*p*=0.026) compared to those of patients without an adverse event, while endothelial nitric oxide synthase levels were similar. Further these results suggest that the SYNTAX score is not related with an increased risk for recurrent myocardial infarction in patients suffering from premature myocardial infarction.

There was no difference in the distribution of common cardiovascular risk factors between patients with and without a recurrent event. The moderate increase of the SIRT1 protein may suggest a compensatory mechanism to protect the people from the detrimental effects of reinfarction. The potential cardioprotective role of SIRT1 was demonstrated in two other recent studies.

In the first study, conducted by our group, SIRT1 protein was increased in cardiac tissue of patients suffering from postoperative atrial fibrillation, likely to compensate the deleterious effects of tissue hypoxia during/after surgery [43].

In one study, SIRT 1 protected the aging heart from contractile dysfunction, mediated through the inhibition of endoplasmic reticulum stress-mediated apoptosis in cardiac-specific sirtuin 1 knockout mouse model [44]. These two results indicate that SIRT 1 acts as a protective protein in different cardiac pathologies, in terms of either counteracting oxidative stress or cardiac dysfunction, developing after myocardial infarction.

## 5. Conclusion

In conclusion, carrying the CC or CG genotypes, additionally the wild-type C allele of the rs7069102 single-nucleotide polymorphism, was associated with an increased risk of premature myocardial infarction, latter affecting SIRT1 and endothelial nitric oxide synthase protein expressions, irrespective of the underlying *SIRT1* genotype.

### 5.1. Study Limitations

The patient number has to be enhanced to determine a clear association between disease phenotype, outcome, and SIRT1 gene variants.

## Figures and Tables

**Table 1:** Clinical characteristics of patients with STEMI.

Clinical characteristics (*n*; %)	Patients with STEMI (*n*=108)
Recurrent myocardial infarction	12 (11.1)
PCI at FU	17 (15.9)
Heart failure development at FU	3 (2.8)
Hospitalization at FU	22 (20.4)
Localisation of MI (anterior/inferior/other) (%)	56 (51.9)/21 (19.4)/31 (28.7)
IRA (LAD/Cx/RCA/others) (%)	56 (51.9)/19 (17.6)/32 (29.6)/1 (0.9)
SYNTAX score	13.6 ± 7.3
Thrombus aspiration without stenting	3 (2.8)
Elective or emergent CABG after MI	6 (5.6)
Thrombolysis therapy	2 (1.9)
Post-CPR	1 (0.9)
LVEF <45%	25 (23.1)

STEMI, ST-elevation myocardial infarction; PCI, percutaneous coronary intervention; FU, follow-up; MI, myocardial infarction; IRA, infarct-related artery; LAD, left anterior descending artery; Cx, circumflex artery; RCA, right coronary artery; CABG, coronary artery bypass graft surgery; CPR, cardiopulmonary resuscitation; LVEF, left ventricular ejection fraction.

**Table 2:** Biochemical characteristics of patients with STEMI and control subjects.

Biochemical parameters	STEMI group *n*=108	Control group *n*=91	*p*
Triglycerides (mg/dl)	195.6 ± 114.3^*∗*^	142.4 ± 128	0.003
LDL cholesterol (mg/dl)	109.9 ± 45.6	118.9 ± 36.0	0.133
HDL cholesterol (mg/dl)	37.4 ± 9.7^*∗*^	49.2 ± 13.9	<0.001
VLDL (mg/dl)	38.7 ± 23.1^*∗*^	30.3 ± 28.7	0.026
TG/HDL	4.8 ± 3.3^*∗*^	0.5 ± 0.5	<0.001
Fasting blood glucose (mg/dl)	136.6 ± 88.1^*∗*^	100.3 ± 35.7	<0.001
Uric acid (mg/dl)	5.3 ± 1.3^*∗*^	4.4 ± 1.3	<0.001

STEMI, ST-elevation myocardial infarction; *n*, number of individuals; statistical evaluation by Student's *t*-test. The results are shown as mean ± standard deviation (SD). HDL, high-density lipoprotein; LDL, low-density lipoprotein; VLDL, very low-density lipoprotein. ^*∗*^*p* < 0.05.

**Table 3:** Comparison of SIRT1, eNOS protein, TAS, TOS, and OSI levels in patients with STEMI and control subjects.

	STEMI group	Control group	*p*
SIRT 1 protein (ng/ml)	1.24 ± 1.07^*∗*^	0.7 ± 0.46	<0.001
eNOS protein (pg/ml)	274.6 ± 313.9^*∗*^	505.9 ± 571.0	0.001
TAS (mmol trolox equiv./L)	1.88 ± 0.34	1.84 ± 0.32	ns
TOS (mmol H_2_O_2_ equiv./L)	13.9 ± 7.4	12.9 ± 5.7	ns
OSI (arbitrary units)	0.78 ± 0.72	0.72 ± 0.32	ns

Statistical evaluation by Student's *t*-test. The results are shown as mean ± standard deviation (SD). ^*∗*^*p* < 0.05. SIRT 1, sirtuin 1; eNOS, endothelial nitric oxide synthase; TAS, total antioxidant status; TOS, total oxidant status; OSI, oxidative stress index; STEMI, ST-elevation myocardial infarction.

**Table 4:** Results of Pearson's correlation between expression level of SIRT1 and other parameters.

	STEMI group		Control group	
	*r*	*p*	*r*	*p*
eNOS protein (pg/ml)	−0.165	0.094	0.147	0.166
TAS (mmol Trolox equiv./L)	−0.139	0.178	−0.101	0.339
TOS (mmol H_2_O_2_ equiv./L)	0.25	0.808	0.007	0.949
OSI	0.053	0.609	0.038	0.720
Age	0.219	0.026^*∗*^	−0.120	0.258
BMI	0.073	0.463	−0.185	0.079

eNOS, endothelial nitric oxide synthase; TAS, total antioxidant status; TOS, total oxidant status; OSI, oxidative stress index; BMI, body mass index; STEMI, ST-elevation myocardial infarction. ^*∗*^*p* < 0.05; *r*, correlation coefficient.

**Table 5:** Distribution of rs7895833 A > G, rs7069102 C > G, and rs2273773 C > T genotypes and alleles in patients with STEMI (ST-elevation myocardial infarction) and control subjects.

	Patients with STEMI		Control subjects		Statistical analysis	
	(%)	(*n*)	(%)	(*n*)	*χ* ^2^	*p*
rs7895833 A > G genotype						
AA	50.5	54	57.1	52	ns	
AG	49.5	53	42.9	39	ns	
GG	na					
Allele						
A	75	161	79	143	ns	
G	25	53	21	39	ns	
rs7069102 C > G genotype						
CC	12	13	11	10		
CG	66.7^*∗*^	72	50.5	46	7.2	0.027
GG	21.3	23	38.5	35	ns	
Allele						
C	45	98	36	66	3.4	0.066
G	55	118	64	116	ns	
rs2273773 C > T genotype						
CC	na					
CT	100	107	100	91		
TT	na					
Allele						
C						
T						

*n*, number of individuals; na, not available. Statistical evaluation by the chi-squared test. ^*∗*^*p* < 0.05. STEMI, ST-elevation myocardial infarction.

**Table 6:** Comparison of rs7895833 A > G, rs7069102 C > G, and rs2273773 C > T SNPs with SIRT1 and eNOS protein levels.

	SIRT1 protein (ng/ml)			eNOS protein (pg/ml)		
	STEMI group	Control group	*p*	STEMI group	Control group	*p*
rs7895833 A > G genotype						
AA	1.28 ± 1.1^*∗*^	0.71 ± 0.49	0.001	274.23 ± 309.46^*∗*^	503.17 ± 682.03	0.031
AG	1.22 ± 0.6^*∗*^	0.69 ± 0.43	0.002	278.18 ± 323.81^*∗*^	518.99 ± 384.52	0.002
GG	na					
rs7069102 C > G genotype						
CC	1.46 ± 0.66^*∗*^	0.70 ± 0.40	0.004	262.40 ± 287.08	416.91 ± 131.84	0.153
CG	1.21 ± 0.70^*∗*^	1.22 ± 0.44	0.002	283.14 ± 350.69^*∗*^	428.06 ± 304.50	0.024
GG	1.23 ± 0.72^*∗*^	0.72 ± 0.48	0.014	255.11 ± 193.49^*∗*^	626.22 ± 819.25	0.014
rs2273773 C > T genotype	na					
CC						
CT						
TT						

eNOS, endothelial nitric oxide synthase; SIRT1, sirtuin 1; STEMI, ST-elevation myocardial infarction. ^*∗*^*p* < 0.05.

**Table 7:** Values of the biochemical parameters in patients carrying the *SIRT1* SNPs.

	rs33 AA			rs 33AG			rs102 CC			rs 102CG			rs102GG		
	STEMI (*n*=54)	CO (*n*=52)	*p*	STEMI (*n*=53)	CO (*n*=39)	*p*	STEMI	CO	*p*	STEMI	CO	*p*	STEMI	CO	*p*
Triglycerides (mg/dl)	210.5 ± 119.37^*∗*^	157.0 ± 110.44	0.021	185.25 ± 108.68^*∗*^	122.06 ± 148.21	0.032	159.0 ± 112.58	192.0 ± 261.98	0.680	200.83 ± 119.97^*∗*^	136.39 ± 103.74	0.005	201.0 ± 95.13^*∗*^	135.0 ± 96.28	0.016
LDL cholesterol (mg/dl)	111.58 ± 51.85	122.30 ± 35.47	0.228	108.06 ± 38.90	114.06 ± 36.76	0.473	108.31 ± 33.22	115.20 ± 41.48	0.662	108.68 ± 40.54	119.73 ± 36.24	0.154	114.81 ± 65.55	118.86 ± 35.24	0.765
HDL cholesterol (mg/dl)	36.80 ± 9.30^*∗*^	47.34 ± 12.07	<0.001	38.0 ± 10.20^*∗*^	51.75 ± 15.95	<0.001	38.67 ± 9.76^*∗*^	60.0 ± 20.92	0.012	37.56 ± 9.63^*∗*^	48.44 ± 13.59	<0.001	35.84 ± 10.20^*∗*^	46.98 ± 10.53	<0.001
VLDL (mg/dl)	41.23 ± 24.44^*∗*^	31.83 ± 22.03	0.044	36.45 ± 21.74	28.07 ± 36.31	0.183	41.23 ± 24.44	31.83 ± 22.03	0.357	39.52 ± 24.36^*∗*^	27.30 ± 20.78	0.008	40.20 ± 19.03^*∗*^	27.59 ± 19.26	0.021
TG/HDL	6.35 ± 4.16^*∗*^	3.69 ± 3.22	0.001	5.99 ± 6.20^*∗*^	2.69 ± 3.47	0.002	4.70 ± 3.81	3.95 ± 6.13	0.352	6.34 ± 5.73^*∗*^	3.29 ± 3.31	0.001	6.34 ± 4.0^*∗*^	3.07 ± 2.24	0.003
Fasting blood glucose (mg/dl)	128.82 ± 74.84	74.38 ± 45.63	0.058	144.26 ± 100.78^*∗*^	92.97 ± 9.47	0.001	132.08 ± 85.50	85.50 ± 13.57	0.145	138.50 ± 96.40^*∗*^	96.61 ± 17.07	0.007	133.03 ± 59.29	107.46 ± 51.98	0.092
Uric acid (mg/dl)	5.25 ± 1.18^*∗*^	4.45 ± 1.23	0.003	5.38 ± 1.38^*∗*^	4.38 ± 1.30	0.002	5.45 ± 1.05	4.63 ± 1.44	0.148	5.35 ± 1.29^*∗*^	4.47 ± 1.26	0.002	5.12 ± 1.44	4.31 ± 1.22	0.057
BMI	28.65 ± 3.27^*∗*^	25.0 ± 3.90	<0.001	28.82 ± 4.77^*∗*^	24.33 ± 3.96	<0.001	29.23 ± 6.71	24.32 ± 5.31	0.071	28.72 ± 3.69^*∗*^	25.15 ± 3.60	<0.001	28.3 ± 3.48	24.25 ± 3.95^*∗*^	<0.001

*n*, number of individuals. Statistical evaluation by Student's *t*-test. The results are shown as mean ± standard deviation (SD). HDL, high-density lipoprotein; LDL, low-density lipoprotein; VLDL, very low-density lipoprotein; BMI, body mass index. ^*∗*^*p* < 0.05.

## Data Availability

Previously reported data (including SIRT 1, eNOS, and oxidative marker analysis in patients with coronary artery disease or atrial fibrillation) were used to support this study and are available at https://doi.org/10.1371/journal.pone.0090428, eCollection 2014; https://doi.org/10.1371/journal.pone.0115339, eCollection 2015; https://doi.org/10.1007/s00431-014-2424-1; https://doi.org/10.1016/j.carpath.2016.02.002. These prior studies (and datasets) are cited at relevant places within the text as references [19, 40, 41, 43].

## Additional Points

(1) This new report highlights *SIRT1* SNPs are associated with premature MI, affecting SIRT1 protein expression. The risk for PMI was increased by 2.31 times in carriers of the CC or CG genotypes of rs7069102 C > G. (2) SIRT1 protein levels were markedly increased in PMI patients compared to the control group, irrespective of the underlying SIRT 1 genotype. SIRT1-induction might be the result of a compensatory mechanism to counteract the adverse effects of oxidative stress in patients with cardiovascular disease. (3) Patients with PMI had a high cardiovascular risk profile, including diabetes (18.5% vs 3.3% *p*=0.001), smoking history (87.1% vs 63.8% *p*=0.727), dyslipidemia (73.1% vs 12.1% *p* < 0.001), and family history for CAD (58.3% vs 30.8 *p* < 0.001). There was no relation detected between cardiovascular risk profile and SIRT 1 SNPs. (4) The concentration of eNOS was considerably low in PMI patients compared to that in the control group (Table 3), whereas no correlation was found between levels of SIRT1 and eNOS (Table 4). Thus, decreased levels of eNOS and increased levels of SIRT 1 might be disease specific, but regulated via different mechanisms. (5) Recurrent MI was observed in 12 cases, and all patients had their first coronary event under the age of 40. SIRT1 protein levels were enhanced compared to the patients without an adverse event, and there was a negative correlation was detected between recurrent MI and left ventricular ejection fraction (LVEF) as well as between SIRT1 values and LVEF. The moderate increase of the SIRT1 protein may suggest a compensatory mechanism to protect the people from the detrimental effects of reinfarction.
